# Craniography

**Published:** 1858-11

**Authors:** 


CRANIOGRAPHY appears to be slowly but surely asserting its claims to be received and acknowledged as a science of some importance. Founded to a great extent upon the well-known works of Blumenbach and Morton, it has, within the past few years, advanced considerably, and assumed a more comprehensive and positive character in consequence of the great additions made to it by the publication of the magnificent Atlas of Dumoutier, the Crania Britannica of Davis and Thurnam, the Deformations du Crâne of Gosse, and the important monographs of Huschke, Virchow, and others in Europe. On this side of the Atlantic, besides the more elaborate works of Morton, various craniographic articles have been published at different times by Mr. J. S. Phillips, Prof. D. Wilson, of Canada, Drs. C. D. Meigs, Neill, Nott, Ramsay, Johnson, and J. Aitken Meigs. The last-named gentleman again directs the attention of the scientific public to this interesting subject, in a small pamphlet, entitled "Hints to Craniographers upon the Importance and Feasibility of Establishing some Uniform System by which the Collection and Promulgation of Craniological Statistics, and the Exchange of Duplicate Crania may be Promoted." In this article, which was read before the Academy of Natural Sciences of this city, at one of its recent meetings, and has since been published in the *Proceedings* of that Society, Dr. M. alludes to the slow progress of craniology, and attributes it chiefly to the want of combination, or some regularly systemized effort among the cultivators of this science. He also speaks of the utility of making cranial collections, enumerates many that have been established, and urges upon their proprietors the importance of printing catalogues of the same, and distributing them to all who may be interested in this branch of the natural history of man. Finally, he says :—

"To the student of craniology, catalogues of all cranial collections, whether large or small—especially if they be descriptive—are of very considerable value. They make known to him the existence of other collections besides his own, and inform him what races and tribes of men are represented therein, or in other words, precisely how much and what available material has been collected for the furtherance of his scientific specialty. He may be desirous of studying the cranial characters of a particular race, of which the specimens in the only collection to which he has access are few in number or of doubtful origin. Having exact catalogues of the various cranial collections which have been made, from time to time, and deposited in different parts of the world, he turns to these and at once learns how many specimens of this race, besides his own, have been collected, and where they are located. He at once opens a correspondence with the proprietors of these collections, and is soon put in possession of any information which he may desire.

"Moreover, these catalogues would form an admirable basis for the interchange of duplicate crania, for the owners of them would know exactly where to apply to make up their deficiencies. The correspondence, also, to which the interchange of catalogues and duplicate crania would give rise, would of itself greatly facilitate the progress of craniography, by making the students of this science acquainted with each other, and enabling them, by a private interchange of opinions, to verify their conclusions or examine them in different points of view before publication. There can hardly be a doubt that the different collections would be respectively increased by the extensive distribution of such catalogues in the hands of army, navy, and other government officials, officers of merchant-men, travelers, naturalists connected with exploring expeditions, and others whose opportunities might be favorable to making such collections, and who would cheerfully do so, were their attention once explicitly directed to this matter.

"Again, the progress of craniography might be very much and very readily facilitated by some such plan or system of co-operation as the following: Let all those actually engaged or interested in the study, in any particular country, notify the secretary, or appropriate officer, of the most prominent and best known scientific institution in that country, of the existence and precise location of any collection of crania with which they may be acquainted, no matter how small or imperfect such a collection may be, stating carefully the name and address of the Society or individual owning the collection, the number of skulls contained, and the different races of men represented therein. Let the secretary or other officer receiving such communications cause them to be published from time to time in the printed journal, transactions or proceedings of the Society. These being sent in exchange or otherwise, to scientific associations and individuals in other countries, would thus become the vehicle for the transmission of this information to the craniographers of the latter places.

"The editors of scientific, medical and literary magazines, journals, reviews, &c., have it in their power greatly to promote craniographic science by inserting in their pages from time to time, and thus disseminating the information obtained in the manner indicated above. This statement particularly applies to medical journals, inasmuch as most of those cultivating craniography are physicians, not a few of whom are in the active public or private practice of their profession."

